# Influence of ad Libitum Feeding of Piglets With *Bacillus Subtilis* Fermented Liquid Feed on Gut Flora, Luminal Contents and Health

**DOI:** 10.1038/srep44553

**Published:** 2017-03-14

**Authors:** Yuyong He, Chunxia Mao, Hong Wen, Zhiyu Chen, Tao Lai, Lingyu Li, Wei Lu, Huadong Wu

**Affiliations:** 1 Jiangxi Province Key Laboratory of Animal Nutrition, Jiangxi Agricultural University, Nanchang 330045, China; 2 Jiangxi Provincial Institute of Veterinary Drugs and Feed Control, Nanchang 330096, China; 3 College of Animal Science and Technology, Jiangxi Agricultural University, Nanchang 330045, China

## Abstract

Some scholars caution that long-term ad libitum feeding with probiotic fermented food poses potential health risks to baby animals. We conducted a feeding experiment to investigate the influence of ad libitum feeding of pre-and post-weaned piglets with a *Bacillus subtilis* fermented diet on the gut microbiome, gut metabolomic profiles, bile acid metabolism, proinflammatory cytokines and faecal consistency. Compared with piglets fed a *Bacillus subtilis*-supplemented pellet diet, piglets fed the *Bacillus subtilis* fermented liquid diet had lower intestinal bacterial diversity (P > 0.05), higher intestinal fungal diversity (P > 0.05), more Firmicutes (P > 0.05), fewer Bacteroidetes, Actinobacteria and Proteobacteria (P > 0.05), higher concentrations of 3-hydroxypropionic acid (P < 0.05), orotic acid (P < 0.05), interleukin-6 (P < 0.01), lactic acid (P < 0.01), deoxycholic acid (P > 0.05) and lithocholic acid (P < 0.01) and a higher incidence of diarrhoea (P > 0.05). The data show that ad libitum feeding of piglets with a *Bacillus subtilis* fermented liquid diet during the suckling and early post-weaning periods promotes the growth of lactic acid bacteria, bile salt hydrolase-active bacteria and 7a-dehydroxylase-active bacteria in the intestinal lumen; disturbs the normal production of lactic acid, orotic acid and unconjugated bile acids; and increases circulating interleukin-6 levels and diarrhoea incidence.

Probiotics have been proven to be useful in rebalancing the intestinal flora, improving inflammation and digestion and preventing cardiovascular diseases^1^,^2^,^3^,^4^, and as a result probiotics are now widely used in food processing and disease control and prevention. To improve the health and growth of children and young animals, specific probiotics are often added to their food at varying doses. Experiments have been conducted to investigate the effects of the routine intake and excessive intake of probiotics on intestinal flora composition, digestion and intestinal health in calves, lambs, piglets and human infants. The resulting data have shown that feeding a moderate dose of probiotics to calves and lambs improves their health and performance^5^,^6^,^7^,^8^,^9^. Kukkonen *et al*. reported thatthe daily feeding of probiotics to newborn human infants for 6 months using an 8–9 × 10^9^ colony-forming-unit mixture of specific probiotics was safe^10^. However, others have argued that supplementing the daily food of infants with probiotics should be done with caution or not at all^11^,^12^,^13^ because of the underdeveloped state of the infant immune system^14^. Li *et al*. (2012) found that oral administration of Lactobacillus rhamnosus at a high dose to piglets resulted in severe diarrhoea^15^. Thus, there is still controversy regarding the safety and impact of probiotics on young animals, particularly regarding the strains, dosage and duration of probiotic administration. These factors should be taken into account as different strains, dosages and durations may have drastically different effects than intended^16^. More information is needed regarding the long-term safety of probiotics and probiotic fermented food^11^,^13^, especially regarding lactic acidosis and bile salt malabsorption caused by bacterial overgrowth; these issues have been rarely studied^11^.

The intestinal flora of pigs plays important roles in intestinal morphology, immunity, digestion and health^17^,^18^,^19^. Generally, from a phylum-level perspective, the flora of the pig intestine can be classified into five phyla: Firmicutes, Bacteroidetes, Proteobacteria, Actinobacteria and Spirochaetes. Firmicutes represents the largest proportion of the total population, followed by Bacteroidetes. These two phyla account for approximately 90% of all the bacteria present in the pig intestine. However, the intestinal microbiota is dynamic, and its composition changes continually in response to time, age, diet, probiotics and many other factors^20^.

In the present study, suckling piglets were used as a model to study the influence of long-term ad libitum feeding of a *Bacillus subtilis* fermented liquid diet on intestinal flora composition, pH, unconjugated bile acids, inflammation and diarrhoea in order to inform risk assessments and investigate the safety of using *Bacillus subtilis* fermented products as a daily food for baby monogastric animals.

## Results

### Operational Taxonomic Unit (OTU) and Alpha Diversity

The sequence data produced in this experiment have been deposited in the National Center for Biotechnology Information (NCBI) Sequence Read Archive (SRA) under accession number SRP060218. Data on OTU and alpha diversity of the microorganism communities in different dietary treatment groups are listed in Table 1 The OTU number, Chao 1 and Shannon values of bacterial communities in the jejunal luminal content of weaned piglets from the *Bacillus subtilis* fermented liquid diet (BFLD) group were lower (*P* > 0.05) than those of weaned piglets from the *Bacillus subtilis*-supplemented commercial pellet diet (BCPD) group. In contrast, the OTU numbers and Chao 1 values of bacterial communities in the colonic luminal content of weaned piglets from the BFLD group were higher (*P* > 0.05) than those of weaned piglets from the BCPD group, which did not hold true for the Shannon value. These results indicated that the feeding of *Bacillus subtilis* fermented liquid diet to piglets decreased bacterial richness and diversity in the jejunal luminal content and decreased bacterial diversity in the colonic luminal content. The Chao 1 values of the fungal community were lower (*P* > 0.05) in the jejunal luminal content but were higher (*P* > 0.05) in the colonic luminal content of weaned piglets from the BFLD group than those of weaned piglets from the BCPD group. These findings suggest that feeding with a *Bacillus subtilis* fermented liquid diet increases fungal diversity in the jejunal and colonic luminal contents of weaned piglets compared with feeding with a *Bacillus subtilis*-supplemented pellet diet.

### Compositions and Relative Abundances of Microorganisms in Jejunal and Colonic Luminal Contents

The compositions and relative abundances of microorganisms in the jejunal and colonic luminal contents are shown in Tables 2 and 3, respectively. Firmicutes and Ascomycota were the dominant phyla in the jejunal and colonic luminal contents of weaned piglets fed either the *Bacillus subtilis* fermented liquid diet or the *Bacillus subtilis*-supplemented commercial pellet diet. Compared with weaned piglets from the BCPD group, weaned piglets from the BFLD group had a higher (*P* > 0.05) relative abundance of Firmicutes in the jejunal and colonic luminal contents, a lower (*P* > 0.05) relative abundance of Ascomycota in the jejunal luminal content and a higher (*P* > 0.05) relative abundance of Ascomycota in the colonic luminal content. The relative abundances of Bacteroidetes, Actinobacteria and Proteobacteria were lower (*P* > 0.05) in the jejunal and colonic luminal contents of weaned piglets from the BFLD group compared with those from the BCPD group. At the genus level, *Lactobacillus* and *Kazachstania* were the dominant genera in jejunal and colonic luminal contents of weaned piglets from both the BFLD and BCPD groups. The relative abundance of *Lactobacillus* in the jejunal and colonic luminal contents of weaned piglets from the BFLD group was higher (*P* > 0.05) than that of weaned piglets from the BCPD group. Weaned piglets from the BFLD group had a lower (*P* > 0.05) relative abundance of *Kazachstania* in the jejunal luminal content and a higher (*P* > 0.05) relative abundance of *Kazachstania* in the colonic luminal content compared with weaned piglets from the BCPD group. The relative abundances of *Streptococcus, Clostridium_sensu_stricto, Bacteroides* and *Flavobacterium* in the jejunal luminal content of weaned piglets from the BFLD group were significantly lower (*P* < 0.01 or *P* < 0.05) than those of weaned piglets from the BCPD group. The relative abundances of *Pseudobutyrivibrio, Lachnospiraceae_unclassified, Erysipelotrichaceae_unclassified, Ruminococcus, Clostridiales_unclassified* and *Lachnospiraceae_uncultured* in the colons of weaned piglets from the BFLD group were significantly higher (P < 0.01 or *P* < 0.05) than those of weaned piglets from the BCPD group.

### Differential Metabolite Levels in Jejunal and Colonic Luminal Contents

All metabolites found at levels that differed between the two piglet groups are listed in Tables 4 and 5. Thirteen differentially observed metabolites in the jejunal luminal content and eleven differentially observed metabolites in the colonic luminal content were identified. Piglets from the BFLD group had higher (*P* < 0.05) relative levels of 3-hydroxypropionic acid and orotic acid in their jejunal luminal content and higher (*P* < 0.05) relative levels of stigmasterol in their colonic luminal content than piglets from the BCPD group. Except for 3-hydroxypropionic acid, orotic acid and stigmasterol, the relative levels of other differentially observed metabolites in the jejunal and colonic luminal contents of weaned piglets from the BFLD group were significantly lower (*P* < 0.01 or *P* < 0.05) than those of piglets from the BCPD group.

### Serum Cytokines, Intestinal pH and Unconjugated Bile Acids

Piglets from the BFLD group had significantly higher serum interleukin-6 (IL-6) levels (*P* < 0.01) than piglets from the BCPD group (Table 6). There were no significant differences (*P* > 0.05) in the levels of serum tumour necrosis factor-alpha (TNF-α) and IL-1β between the BFLD and BCPD groups.

There was no significant difference in the pH values of the jejunal luminal content between the BFLD and BCPD groups (*P* > 0.05). However, the pH values of the colonic luminal content collected from piglets in the BFLD group were significantly lower than the pH values of the colonic luminal content collected from piglets in the BCPD group (*P* < 0.05).

The jejunal luminal content collected from the BFLD group had significantly higher lactic acid and lithocholic acid (LCA) concentrations (*P* < 0.01) and significantly lower cholic acid (CA) concentrations (*P* < 0.05) than that collected from the BFLD group. There were no significant differences in chenodeoxycholic acid (CDCA) and deoxycholic acid (DCA) concentrations in the jejunal luminal content between the BFLD and BCPD groups (*P* > 0.05).

The colonic luminal content collected from the BFLD group had significantly higher lactic acid, chenodeoxycholic acid and lithocholic acid concentrations than that collected from the BCPD group (*P* < 0.01). The concentrations of cholic acid and deoxycholic acid in the colonic luminal content collected from the BFLD group were not significantly higher than those in the colonic content collected from the BCPD group (*P* > 0.05).

### Diarrhoea Incidence

The data in Table 7 show that piglets from the BFLD group had a higher incidence of diarrhoea than piglets from the BCPD group at each experimental time point, but there was no significant difference (*P* > 0.05) in the incidence of diarrhoea between the BFLD and BCPD groups.

## Discussion

Previous studies have reported that the diversity, composition and relative abundance of intestinal flora can be influenced by probiotic administration or dietary patterns^21^,^22^. The feeding of probiotics and a probiotic fermented diet to animals decreases microbial diversity, and the reduced microbial diversity is often associated with gastrointestinal disorders, including inflammatory bowel disease^23^. In the present study, 25-day feeding with a *Bacillus subtilis* fermented liquid diet to piglets aged 7 to 31 days decreased the bacterial diversity but increased the fungal diversity of jejunal and colonic luminal contents compared with 25-day feeding with a *Bacillus subtilis*-supplemented pellet diet; the decreased bacterial diversity of piglets fed a *Bacillus subtilis* fermented liquid diet resulted in a higher diarrhoea incidence than observed in the piglets fed a *Bacillus subtilis*-supplemented pellet diet. In addition, Ley *et al*. reported that the gut microbiome is dominated by four bacterial phyla: Firmicutes, Bacteroidetes, Actinobacteria and Proteobacteria^24^; data in this study also indicated that the flora in the jejunal and colonic luminal contents of piglets fed continuously with a *Bacillus subtilis* fermented liquid diet or a *Bacillus subtilis-*supplemented pellet diet were also dominated by Firmicutes, Bacteroidetes, Actinobacteria, Proteobacteria and Ascomycota. However, different dietary patterns influenced the relative abundances of intestinal flora: piglets fed a *Bacillus subtilis* fermented liquid diet had higher (*P* > 0.05) relative abundances of organisms from the Firmicutes phylum and *Lactobacillus* genus in their jejunal luminal contents than piglets fed a *Bacillus subtilis*-supplemented pellet diet. Regarding the relative abundances of flora in the colonic luminal contents, piglets fed a *Bacillus subtilis* fermented liquid diet not only had higher (*P* > 0.05) relative abundances of organisms of the Firmicutes phylum and *Lactobacillus* genus but also had higher (*P* > 0.05) relative abundances of organisms of the Ascomycota phylum and *Kazachstania* genus than did piglets fed a *Bacillus subtilis*-supplemented pellet diet. These results indicate that a *Bacillus subtilis* fermented diet has an advantage in promoting the growth of the flora noted above because the *Bacillus subtilis* fermented liquid diet has ingredients more suitable for the growth of intestinal flora than the *Bacillus subtilis*-supplemented pellet diet.

Orotic acid is often regarded as one of the major oxidative stressors at high concentrations^25^. Additionally, circulating levels of IL-6 and the growth of *Coprococcus, Pseudobutyrivibrio* and *Dorea* increase under the action of stressors^26^. Piglets from the BFLD group had significantly higher orotic acid levels in their jejunal luminal content than piglets from the BCPD group. As a result, piglets from the BFLD group had higher circulating IL-6 levels and higher relative abundances of *Coprococcus, Pseudobutyrivibrio* and *Dorea* than piglets from the BCPD group.

Elevated levels of circulating IL-6 are often associated with a number of diseases^27^. People with high IL-6 levels have a high risk of systemic mastocytosis^28^, and elevated circulating IL-6 has been proposed as a marker of inflammation linking obesity with insulin resistance and diabetes as well as atherosclerosis^29^,^30^. High serum levels of IL-6 may also be associated with ankylosing spondylitis in young people, which is characterized by intense joint pain, stiffness, weakness, marasmus and apocleisis^31^.

Studies have demonstrated that diarrhoea can be prevented by the administration of probiotics or probiotic fermented food^32^. However, Li *et al*. (2012) found that oral administration of *Lactobacillus rhamnosus* at a high dose to piglets caused diarrhoea^15^. Data in the present study also showed that piglets from the BFLD group had a higher incidence of diarrhoea than piglets from the BCPD group.

Conjugated bile acids have emulsifying and surfactant properties; they are more efficient than unconjugated bile acids in aiding in the emulsification of dietary lipids and preventing small intestinal bacterial overgrowth^33^,^34^. In normal conditions, the composition of bile acids in the intestine is often in a relative balance; only small amounts of conjugated bile acids are hydrolyzed into primary bile acids (CA and CDCA), and small amounts of primary bile acids are dehydroxylated into secondary bile acids (DCA and LCA) in the small intestine^35^. Approximately 95% of bile acids (conjugated and unconjugated) are reabsorbed by the distal ileum. The small percentage of bile acids remaining reaches the colon, where they are deconjugated and dehydroxylated by bacteria to produce the secondary bile acids (DCA and LCA)^36^. However, overgrowth of bile salt hydrolase-active and 7a-dehydroxylase-active bacteria in the intestine will alter the normal bile acid composition and damage normal enterohepatic circulation.

Some strains of Lactobacillus^37^, Erysipelotrichaceae^38^, Lachnospiraceae^39^, Clostridium^40^,^41^ and Bacteroides^42^,^43^ are bile salt hydrolase-active intestinal bacteria, and some strains of Lactobacillus^44^, Lachnospiraceae^45^,^46^, Ruminococcaceae^45^, Clostridiaceae^46^,^47^, Eubacterium^48^ and Peptostreptococcus^49^ are 7a-dehydroxylase-active intestinal bacteria. In the present study, compared with piglets from the BCPD group, piglets from the BFLD group had lower (P < 0.01) relative abundances of Clostridium and Bacteroides in their jejunal luminal content. This condition resulted in piglets from the BFLD group having lower CA and CDCA levels in their jejunal luminal content. *Lactobacilli* are also 7a-dehydroxylase-active bacteria, and the relative abundance of *Lactobacilli* in the jejunal luminal content of piglets from the BFLD group were higher than those in piglets from the BCPD group. This is the reason why piglets from the BFLD group had higher DCA and LCA levels in their jejunal luminal content than piglets from the BCPD group did. Piglets from the BFLD group also had higher relative abundances of Lactobacillus, Ruminococcaceae, Lachnospiraceae, Erysipelotrichaceae, Ruminococcus and Clostridiales in their colonic luminal contents than piglets from the BCPD group. Therefore, in the BFLD piglets, more conjugated bile acids were hydrolyzed into CA and CDCA, while CA and CDCA were dehydroxylated into DCA and LCA, respectively. As a result, piglets from the BFLD group had higher CA, CDCA, DCA and LCA levels in their colonic luminal content than piglets from the BCPD group.

Unconjugated bile acids are less water soluble than conjugated bile acids. Intense elevation of the concentration of unconjugated bile acids has detrimental effects on the intestinal mucosa, including mucosal damage, increased mucosal permeability and potentially colon cancer-promoting effects^50^,^51^,^52^. Increased CDCA and DCA levels can inhibit water absorption and induce water and sodium secretion by the colon at concentrations above 3 mmol/L^53^ and can disturb the normal microbiota of the gut, leading to diarrhoea and mucosal inflammation in the intestinal contents^54^. Piglets from the BFLD group had higher CDCA and DCA in their colonic luminal contents than piglets from the BCPD group; this is one of the factors contributing to the higher diarrhoea incidence among piglets from the BFLD group.

The *Bacillus subtilis* fermented liquid diet contains more active components for the growth of lactic acid bacteria and a high lactic acid concentration^55^,^56^. These active components allowed lactic acid-producing bacteria to grow better in the intestines of piglets from the BFLD group than in the intestines of piglets from the BCPD group. The higher relative abundance of lactic acid bacteria together with the high lactic acid intake resulted in piglets from the BFLD group having higher lactic acid in their jejunal and colonic luminal contents than piglets from the BCPD group. Excessive lactic acid in the intestine often causes lactic acidosis, which can induce diarrhoea^57^. Thus, lactic acidosis is the other factor contributing to the higher diarrhoea incidence among piglets from the BFLD group than among piglets from the BCPD group.

In summary, ad libitum feeding of pre-and post-weaned piglets with a *Bacillus subtilis* fermented liquid diet decreased intestinal bacterial diversity and increased intestinal fungal diversity, circulating IL-6 levels, intestinal unconjugated bile acid concentrations and diarrhoea incidence. Lactic acidosis, dietary lipid malabsorption and the inducing effect of unconjugated bile salts are the underlying causes for the higher diarrhoea incidence among piglets fed the *Bacillus subtilis* fermented liquid diet.

## Materials and Methods

### Animal Treatment Protocol

Twelve lactating sows (Large White x Landrace, artificially inseminated with semen from one Duroc boar) with similar body conditions and suckling piglets were randomly assigned to one experimental group and one control group (6 lactating sows +56 suckling piglets vs 6 lactating sows +54 suckling piglets) at the 7^th^ day after farrowing. There was no significant difference (*P* > 0.05) in the average body weight (2.79 ± 0.19 kg/piglet vs 2.85 ± 0.27 kg/piglet) of suckling piglets at 7 days of age between the experimental and control groups. All lactating sows were fed the same commercial lactation diet (7.5 kg/d). Piglets in the experimental group and control group had free access to a *Bacillus subtilis* fermented liquid diet (live *Bacillus subtilis*: 12.75 × 10^8^ CFU/g) or a *Bacillus subtilis*-supplemented commercial pellet diet (live *Bacillus subtilis*: 2.80 × 10^8^ CFU/g), respectively, from 7 to 31 days of age. All suckling piglets were weaned at 21 days of age. The *Bacillus subtilis* fermented liquid diet was produced using a previously described method^46^.

A total of six piglets (each with a body weight closest to the average weight of the litter) in each dietary treatment group (3 males and 3 females) were slaughtered in the morning at 32 days of age according to the protocol approved by the Animal Ethics Committee of Jiangxi Agricultural University.

### Sample Collection

Before slaughter, blood was collected with a 10 mL fresh tube from the jugular vein. Serum was separated by centrifugation after blood clotting and stored at −20 °C for the analysis of serum cytokines.

After slaughter, the segments of the jejunum and colon were quickly excised. The contents of the jejunum and colon were separately collected with 10 mL fresh tubes and immediately stored at −80 °C for the analysis of pH, lactic acid, unconjugated bile acids, microbial composition and differential metabolites.

### pH Measurement

A digital pH metre (LP115FK, Mettler Toledo, Switzerland) was used to measure the pH of samples after calibration with standard buffers (pH 4.0 and 7.0).

### Enzyme-linked Immunosorbent Assay

The concentrations of interleukin −1β, interleukin −6 and tumour necrosis factor-alpha were determined in three replicates for each sample using enzyme-linked immunosorbent assay kits (R&D Systems, Nanjing Jiancheng Bioengineering Institute).

Concentrations of D-/L-lactic acid, cholic acid, chenodeoxycholic acid, deoxycholic acid and lithocholic acid in the intestinal contents were determined in three replicates for each sample using enzyme-linked immunosorbent assay kits (R&D Systems, Shanghai Enzyme-linked Biotechnology Co., Ltd.).

### Microbiome and Metabolomics Analysis

Genomic DNA of each sample was extracted using the E.Z.N.A. Soil DNA kit (OMEGA, USA), and six genomic DNA preparations for each treatment group were pooled into three DNA mixtures prior to polymerase chain reaction (PCR).

Bacterial genomic DNA was amplified with primers covering the V1–V3 region of the 16 S rRNA bacterial gene; the bar-coded primers 27 F and 533 R containing A and B sequencing adaptors (454 Life Sciences) were used. The forward primer (B-27F) was 5′-CCTATCCCCTGTGTGCCTTGGCAGTCGACTAGAGTTTGATCCTGGCTCAG-3′; the sequence of the B adaptor is shown in italics and is underlined. The reverse primer (A-533R) was 5′-CCATCTCATCCCTGCGTGTCTCCGACGACTNNNNNNNNNNTTACCGCGGCTGCTGGCAC-3′; the sequence of the A adaptor is shown in italics and underlined, and the Ns represent a ten-base sample specific barcode sequence^58^.

Fungal genomic DNA was amplified using the forward primer (A-ITS1) and reverse primer (B-ITS4). The forward primer (A-ITS1) was 5′-CCATCTCATCCCTGCGTGTCTCCGACGACTNNNNNNNNNNTCCGTAGGTGAACCTGCGG-3′; the sequence of adaptor A is shown in italics and underlined, and the Ns represent a ten-base sample specific barcode sequence. The reverse primer (B-ITS4) was 5′-CCTATCCCCTGTGTGCCTTGGCAGTCGACTTCCTCCGCTTATTGATATGC-3′, and the sequence of adaptor B is shown in italics and underlined.

The protocols for PCR, pyrosequencing, sequence processing and bioinformatic analyses were previously described^56^,^59^. Differential metabolites were determined using gas chromatography time of flight mass spectrometry^55^.

### Diarrhoea Incidence Calculation

Faecal consistency was visually examined at the same time each morning by the same person during experimental periods. A piglet was considered to have diarrhoea when it developed a pasty or watery faecal consistency. Diarrhoea incidence was defined as the percentage of animals with diarrhoea on a specific day^60^.

### Statistical Analysis

Data analysis was performed with SPSS software (version 13.0). One-way analysis of variance (ANOVA) was used to evaluate significant differences between means with a significance level at α = 0.01. Tukey’s test was used to perform comparisons between means. Data were presented as the means ± SEM.

## Additional Information

**How to cite this article**: He, Y. *et al*. Influence of ad libitum feeding of piglets with *Bacillus subtilis* fermented liquid feed on gut flora, luminal contents and health. *Sci. Rep.* **7**, 44553; doi: 10.1038/srep44553 (2017).

**Publisher's note:** Springer Nature remains neutral with regard to jurisdictional claims in published maps and institutional affiliations.

## Figures and Tables

**Table 1:** Results of OTU, species richness and diversity of microorganism communities.

	BFLD group	BCPD group	p-value
Bacteria: samples of jejunal luminal content			
OTU	74.00 ± 5.77	117.00 ± 34.07	0.281
Chao 1	101.00 ± 11.72	138.00 ± 29.21	0.305
Shannon	1.06 ± 0.18	2.07 ± 0.51	0.133
Bacteria: samples of colonic luminal content			
OTU	180.00 ± 11.27	137.67 ± 31.87	0.279
Chao 1	219.33 ± 5.61	168.67 ± 29.85	0.171
Shannon	2.03 ± 0.19	2.50 ± 0.38	0.324
Fungi: samples of jejunal luminal content			
OTU	24.33 ± 1.20	29.33 ± 2.91	0.187
Chao 1	25.33 ± 1.20	31.00 ± 2.89	0.144
Shannon	1.41 ± 0.10	1.28 ± 0.19	0.578
Fungi: samples of colonic luminal content			
OTU	24.00 ± 1.15	25.67 ± 3.84	0.699
Chao 1	26.67 ± 0.88	26.33 ± 4.18	0.942
Shannon	1.28 ± 0.02	1.27 ± 0.22	0.955

**Table 2:** Compositions and relative abundance of microorganism in jejunal luminal content.

Phylum level	Genus level	Relative abundance of samples in jejunal luminal content		
		BFLD group	BCPD group	p value
Bacterial community				
Firmicutes		99.19 ± 0.57	90.12 ± 9.41	0.390
	Lactobacillus	97.81 ± 1.13	63.84 ± 31.07	0.172
	Lactococcus	0.61 ± 0.39	11.55 ± 11.14	0.679
	Bacillus	0.16 ± 0.10	4.78 ± 4.65	0.666
	Streptococcus	0.14 ± 0.03	3.76 ± 1.40	0.005
	Solibacillus	0.10 ± 0.06	2.64 ± 2.56	0.670
	Enterococcus	0.08 ± 0.08	0.65 ± 0.40	0.092
	Exiguobacterium	0.03 ± 0.02	0.45 ± 0.44	0.737
	Leuconostoc	0.02 ± 0.01	0.15 ± 0.14	0.712
	Lysinibacillus	0.02 ± 0.01	0.40 ± 0.38	0.664
	Peptostreptococcaceae_uncultured	0.02 ± 0.01	0.16 ± 0.08	0.047
	Brochothrix	0.01 ± 0.01	0.13 ± 0.12	0.731
	Carnobacterium	0.00 ± 0.00	0.19 ± 0.19	0.258
Bacteroidetes		0.01 ± 0.01	0.21 ± 0.11	0.128
	Bacteroides	0.00 ± 0.00	0.02 ± 0.01	0.012
	Flavobacterium	0.00 ± 0.00	0.03 ± 0.01	0.020
Actinobacteria		0.11 ± 0.04	1.69 ± 1.46	0.338
	Arthrobacter	0.05 ± 0.02	1.35 ± 1.32	0.669
	Propionibacterium	0.01 ± 0.01	0.11 ± 0.10	0.226
Proteobacteria		0.18 ± 0.12	1.35 ± 1.33	0.432
	Escherichia-Shigella	0.11 ± 0.09	0.30 ± 0.30	0.881
	Pelomonas	0.01 ± 0.00	0.14 ± 0.13	0.676
	Pseudomonas	0.01 ± 0.01	0.14 ± 0.14	0.759
Fungal community				
Ascomycota		96.17 ± 3.75	98.29 ± 1.08	0.616
	Kazachstania	95.81 ± 3.67	97.2 ± 1.85	0.851
	Chrysosporium	0.13 ± 0.13	0.05 ± 0.05	0.750
	Candida	0.13 ± 0.05	0.23 ± 0.17	0.714
Fungi_unclassified		3.79 ± 3.72	1.65 ± 1.09	0.721
	Fungi_unclassified	3.79 ± 3.72	1.65 ± 1.09	0.721

**Table 3:** Compositions and relative abundance of microorganism in colonic luminal content.

Phylum level	Genus level	Relative abundance of samples in colonic luminal content		
		BFLD group	BCPD group	p value
Bacterial community				
Firmicutes		97.25 ± 0.35	87.45 ± 6.39	0.201
	Lactobacillus	78.90 ± 5.05	43.82 ± 23.82	0.124
	Ruminococcaceae_uncultured	8.17 ± 3.75	3.38 ± 1.80	0.240
	Blautia	1.33 ± 0.65	0.62 ± 0.40	0.593
	Ruminococcaceae_incertae_sedis	1.14 ± 0.76	0.24 ± 0.19	0.234
	Roseburia	0.93 ± 0.72	0.09 ± 0.07	0.239
	Subdoligranulum	0.86 ± 0.32	1.30 ± 0.69	0.816
	Erysipelotrichaceae_incertae_sedis	0.77 ± 0.30	0.92 ± 0.37	0.917
	Pseudobutyrivibrio	0.66 ± 0.15	0.03 ± 0.03	0.008
	Lachnospiraceae_unclassified	0.58 ± 0.10	0.23 ± 0.13	0.028
	Erysipelotrichaceae_unclassified	0.47 ± 0.20	0.00 ± 0.00	0.020
	Ruminococcus	0.37 ± 0.18	0.01 ± 0.01	0.039
	Dorea	0.30 ± 0.04	0.18 ± 0.09	0.213
	Ruminococcaceae_unclassified	0.30 ± 0.16	0.37 ± 0.19	0.935
	Anaerotruncus	0.25 ± 0.12	0.37 ± 0.18	0.836
	Faecalibacterium	0.23 ± 0.08	2.12 ± 1.86	0.295
	Coprococcus	0.18 ± 0.17	0.04 ± 0.04	0.681
	Clostridiales_unclassified	0.17 ± 0.07	0.01 ± 0.01	0.019
	Erysipelotrichaceae_uncultured	0.15 ± 0.07	0.04 ± 0.02	0.087
	Erysipelotrichaceae_norank	0.13 ± 0.07	0.05 ± 0.04	0.269
	Flavonifractor	0.12 ± 0.10	0.30 ± 0.21	0.692
	Lachnospiraceae_incertae_sedis	0.12 ± 0.04	0.04 ± 0.03	0.133
	Lachnospiraceae_uncultured	0.10 ± 0.03	0.00 ± 0.00	0.014
	Christensenellaceae_uncultured	0.08 ± 0.02	0.11 ± 0.11	0.941
	Streptococcus	0.06 ± 0.02	6.13 ± 3.99	0.094
	Defluviitaleaceae_incertae_sedis	0.03 ± 0.01	0.35 ± 0.26	0.183
	Enterococcus	0.02 ± 0.01	25.35 ± 25.32	0.310
	Mogibacterium	0.01 ± 0.01	0.08 ± 0.04	0.029
Bacteroidetes		1.17 ± 0.36	8.84 ± 7.43	0.361
	Prevotellaceae_uncultured	0.18 ± 0.16	1.04 ± 0.92	0.612
	Bacteroides	0.10 ± 0.06	0.16 ± 0.15	0.893
	Prevotella	0.08 ± 0.02	5.77 ± 5.22	0.262
	Alloprevotella	0.04 ± 0.04	0.57 ± 0.56	0.598
Actinobacteria		0.32 ± 0.13	1.26 ± 0.43	0.103
	Collinsella	0.28 ± 0.12	1.11 ± 0.44	0.047
Proteobacteria		0.43 ± 0.17	2.19 ± 1.51	0.311
	Campylobacter	0.25 ± 0.22	0.04 ± 0.04	0.595
	Escherichia-Shigella	0.15 ± 0.12	1.57 ± 1.48	0.589
	Leeia	0.00 ± 0.00	0.42 ± 0.41	0.297
	Morganella	0.00 ± 0.00	0.14 ± 0.14	0.518
Fungal community				
Ascomycota		99.88 ± 0.03	99.50 ± 0.16	0.082
	Kazachstania	99.48 ± 0.09	98.06 ± 1.17	0.201
	Cladosporium	0.11 ± 0.01	0.12 ± 0.06	0.885
	Candida	0.07 ± 0.05	0.44 ± 0.25	0.097
	Saccharomycetales_unclassified	0.02 ± 0.01	0.83 ± 0.83	0.556
Fungi_unclassified		0.06 ± 0.01	0.43 ± 0.19	0.047
	Fungi_unclassified	0.06 ± 0.01	0.43 ± 0.19	0.047

**Table 4:** Differential metabolites in jejunal luminal content of weaned piglets between different treatments.

Metabolites	R.T.	Mass	Differential metabolites in jejunal luminal content		VIP	p-value	Fold change
			BFLD group	BCPD group			
Fucose	16.08	117	0.0218 ± 0.00	0.1005 ± 0.03	1.86	0.034	0.22
2-hydroxybutanoic acid	8.26	131	0.0089 ± 0.00	0.0645 ± 0.02	2.03	0.017	0.14
3-hydroxypropionic acid	8.51	177	0.0133 ± 0.00	0.0039 ± 0.00	2.00	0.008	3.41
Glycine	8.20	102	0.1204 ± 0.06	0.5064 ± 0.14	1.75	0.028	0.24
Ornithine	16.26	174	0.0150 ± 0.00	0.0487 ± 0.01	2.13	0.012	0.31
Beta-Alanine	12.42	248	0.0108 ± 0.00	0.0193 ± 0.00	2.09	0.005	0.56
Orotic acid	16.14	254	0.0031 ± 0.00	0.0002 ± 0.00	1.93	0.027	15.50
Pipecolinic acid	11.60	156	0.0080 ± 0.00	0.0202 ± 0.00	1.92	0.022	0.40
Spermidine	20.62	200	0.0019 ± 0.00	0.0068 ± 0.00	2.54	0.000	0.28
Putrescine	16.11	174	0.1229 ± 0.03	0.5455 ± 0.13	1.95	0.023	0.23
N-Acetyl-D-galactosamine	19.51	87	0.0108 ± 0.00	0.0198 ± 0.00	1.86	0.017	0.55
Lignoceric acid	25.59	117	0.0087 ± 0.00	0.0235 ± 0.01	1.68	0.038	0.37
Arachidic acid	22.66	117	0.0195 ± 0.01	0.0568 ± 0.01	1.80	0.023	0.34

**Table 5:** Differential metabolites in colonic luminal content of weaned piglets between different treatments.

Metabolites	R.T.	Mass	Differential metabolites in colonic luminal content		VIP	p-value	Fold change
			BFLD group	BCPD group			
D-Glyceric acid	11.11	189	0.0137 ± 0.00	0.0392 ± 0.01	2.20	0.020	0.35
Melibiose	25.68	204	0.0010 ± 0.00	0.0062 ± 0.00	2.01	0.041	0.16
Sucrose	24.10	361	0.0017 ± 0.00	0.0045 ± 0.00	1.92	0.034	0.38
Gluconic acid	18.76	333	0.0004 ± 0.00	0.0015 ± 0.00	2.47	0.002	0.27
Succinic acid	10.92	147	2.2537 ± 0.66	4.9267 ± 0.97	1.83	0.047	0.46
Pyruvic acid	7.16	174	0.0148 ± 0.01	0.0801 ± 0.02	2.05	0.035	0.18
Glutamic acid	14.77	246	0.3112 ± 0.07	0.6594 ± 0.10	2.10	0.018	0.47
Beta-Alanine	12.42	248	0.0171 ± 0.00	0.0362 ± 0.00	2.09	0.018	0.47
Aspartic acid	12.37	160	0.0145 ± 0.00	0.0479 ± 0.01	2.12	0.030	0.30
Oxoproline	13.66	156	6.8206 ± 1.26	11.9747 ± 1.22	2.14	0.015	0.57
Stigmasterol	28.81	255	0.4551 ± 0.09	0.1490 ± 0.08	1.93	0.033	3.05

**Table 6:** Differences in serum cytokine, pH, lactic acid, total bile acids and unconjugated bile acids between BFLD group and BCPD group.

	BFLD group	BCPD group	p-value
Level of serum cytokine (ng/L)			
IL-1β	19.45 ± 0.85	19.89 ± 0.99	0.731
IL-6	78.87 ± 5.25	56.64 ± 2.59	0.001
TNF-α	86.44 ± 4.91	84.58 ± 4.19	0.775
Jejunal luminal content			
pH	6.79 ± 0.21	6.66 ± 0.24	0.693
Lactic acid (μg/g)	5.21 ± 0.12	1.32 ± 0.09	0.000
Cholic acid (ng/g)	6.36 ± 0.65	8.62 ± 0.43	0.016
Chenodeoxycholic acid (ng/g)	8.43 ± 0.22	8.82 ± 0.43	0.435
Deoxycholic acid (ng/g)	3.99 ± 0.36	3.56 ± 0.30	0.387
Lithocholic acid (ng/g)	2.03 ± 0.13	0.71 ± 0.06	0.000
Colonic luminal content			
pH	6.04 ± 0.14	6.92 ± 0.25	0.012
Lactic acid (μg/g)	6.98 ± 0.20	5.43 ± 0.12	0.000
Cholic acid (ng/g)	2.89 ± 0.30	2.60 ± 0.17	0.432
Chenodeoxycholic acid (ng/g)	1.01 ± 0.04	0.58 ± 0.04	0.000
Deoxycholic acid (ng/g)	4.82 ± 0.43	4.57 ± 0.46	0.695
Lithocholic acid (ng/g)	1.35 ± 0.10	0.56 ± 0.04	0.000

**Table 7:** Difference in diarrhoea incidence between BFLD group and BCPD group.

	BFLD group	BCPD group	p-value
Pre-weaning (d7-d21)	15.74 ± 4.97	11.12 ± 2.97	0.443
Post-weaning (d22-d31)	22.79 ± 3.11	13.87 ± 3.48	0.085
Pre and Post-weaning (d7-d31)	18.67 ± 3.93	12.26 ± 2.97	0.213
